# Cryptic species conservation: a review

**DOI:** 10.1111/brv.13139

**Published:** 2024-09-05

**Authors:** Daniel Hending

**Affiliations:** ^1^ Department of Biology University of Oxford 11a Mansfield Road Oxford OX1 3SZ UK

**Keywords:** applied conservation, biodiversity, conservation challenge, holistic monitoring, morphology, protected areas, remote sensing, speciation

## Abstract

Cryptic species are groups of two or more taxa that were previously classified as single nominal species. Being almost morphologically indistinguishable, cryptic species have historically been hard to detect. Only through modern morphometric, genetic, and molecular analyses has the hidden biodiversity of cryptic species complexes been revealed. Cryptic diversity is now widely acknowledged, but unlike more recognisable, charismatic species, scientists face additional challenges when studying cryptic taxa and protecting their wild populations. Demographical and ecological data are vital to facilitate and inform successful conservation actions, particularly at the individual species level, yet this information is lacking for many cryptic species due to their recent taxonomic description and lack of research attention. The first part of this article summarises cryptic speciation and diversity, and explores the numerous barriers and considerations that conservation biologists must navigate to detect, study and manage cryptic species populations effectively. The second part of the article seeks to address how we can overcome the challenges associated with efficiently and non‐invasively detecting cryptic species *in‐situ*, and filling vital knowledge gaps that are currently inhibiting applied conservation. The final section discusses future directions, and suggests that large‐scale, holistic, and collaborative approaches that build upon successful existing applications will be vital for cryptic species conservation. This article also acknowledges that sufficient data to implement effective species‐specific conservation will be difficult to attain for many cryptic animals, and protected area networks will be vital for their conservation in the short term.

## I. INTRODUCTION

### (1) What are cryptic species?

Cryptic species can be broadly defined as two or more species that have historically been, or are currently classified as the same species due to morphological similarities that make them visually indistinguishable (Bickford *et al*., 2007). Despite the acceptance and use of this definition by many scholars, other definitions and descriptions of cryptic species are persistently used in publications, and there has been considerable debate on how best to describe cryptic species (Jörger & Schrödl, 2013). Some authors have expanded upon the broad definition provided above, and have defined cryptic species as morphological and physiologically similar taxa that are on different evolutionary trajectories (Struck *et al*., 2018). Further, some scholars only recognise a group of morphologically similar taxa as cryptic if they have only recently diverged (Fišer, Robinson & Malard, 2018), are reproductively isolated (Kordbacheh, Rahimian & Fontaneto, 2023), can only be distinguished genetically or molecularly (DeSalle, Egan & Siddall, 2005), or occur sympatrically in the wild (Stebbins, 1950). The ambiguity of the term cryptic species is arguably driven by differing views on what actually constitutes a species, and the ongoing debate about the biological species concept (Donoghue, 1985; Mayr, 1996; Noor, 2002; Wang *et al*., 2020).

To confuse the situation further, cryptic species are commonly referred to as “sibling species” within the published literature (Sáez & Lozano, 2005; Pfenninger & Schwenk, 2007). Whilst many now argue that the term sibling species is applicable only to closely related taxa with recent common ancestry (Knowlton, 1993; Baillie, Hilton‐Taylor & Stuart, 2004), both terms are frequently confused (Borkin *et al*., 2004; Fišer *et al*., 2018). The additional term “sister species” adds further perplexity to our collective understanding of cryptic species complexes, although this phylogenetic term refers specifically to two taxa that are the most closely related in relation to other closely related species as they are derived from the same immediate common ancestor (Bickford *et al*., 2007; Pontarp, Ripa & Lundberg, 2015). Notably, the term “cryptic” itself also has two different meanings in biological studies, with a species, most often an animal, sometimes referred to as cryptic if it is elusive, secretive or otherwise hard to detect and study within its natural habitat (e.g. Claridge *et al*., 2004; Aubry, Raley & McKelvey, 2017). Herein, I use a broad definition and define cryptic species as species that have been, or are currently, classified as single species due to their near‐identical morphological appearance, and if already described, are still very hard or impossible to tell apart by traditional identification means (i.e. morphology or visual appearance). To avoid further confusion in an already complex problem, I also do not discuss sub‐species in this review.

### (2) Mechanisms causing cryptic diversification

#### (a) Drivers of speciation and cryptic complexes

Cryptic species complexes are the result of recent and sometimes high‐rate speciation (Gómez *et al*., 2002), but the diversification of cryptic species can be driven by a wide range of biogeographic, phenotypic and climatic processes (Miranda *et al*., 2022). Cryptic speciation is often the result of landscape fragmentation (Smith *et al*., 2014), where natural barriers prevent gene flow between subpopulations causing reproductive isolation and subsequently speciation (Li *et al*., 2014). Such natural barriers include rivers, oceans, hills, mountain chains, and deserts, all of which can result in allopatric speciation among isolated subpopulations of taxa (Wilmé, Goodman & Ganzhorn, 2006; Britton, Hedderson & Verboom, 2014; Miranda *et al*., 2022). However, more‐recent landscape fragmentation due to anthropogenic factors can also be the cause of cryptic speciation. Such examples include deforestation, agricultural expansion, and even the establishment of large man‐made structures (Su *et al*., 2003; Huntley *et al*., 2019). Whilst arguably less discussed in the literature, climatic changes resulting in bioclimatic zonation also drive speciation and the formation of cryptic species complexes (Kozak & Wiens, 2007), although bioclimatic zonation is often strongly linked to biogeography [e.g. Madagascar (Wilmé *et al*., 2006; Vences *et al*., 2009; Brown *et al*., 2016)]. Areas with high levels of bioclimatic and biogeographic zonation are therefore hotspots for cryptic species complexes (Kozak & Wiens, 2007). Natural and sexual selection also play a role in speciation (Jones, 1997), examples of which include divergences of sexual behaviour (Pillay & Rymer, 2012), acoustic signals (Hasiniaina *et al*., 2020), pheromones (Zozaya *et al*., 2019) and facial structure (Chornelia, Lu & Hughes, 2022). Diversification of these phenotypes have resulted in cryptic species complexes of genetically distinct, yet morphologically indistinguishable species, that are most easily identified by variation in the aforementioned phenotypes.

#### (b) Causes of morphological “identicalness”

Morphological indistinguishability among cryptic species can be the result of a number of different mechanisms. Fišer *et al*. (2018) highlighted three primary hypotheses that postulate why cryptic species possess near‐identical morphologies making it very difficult visually to discriminate among them. Firstly, the recent divergence hypothesis suggests that morphological discrepancies are not yet present among closely related cryptic species due to their recent divergence (Egea *et al*., 2016). Second, the phylogenetic niche conservatism hypothesis points to morphological stasis, where morphological differentiation is constrained among closely related cryptic species by selection (Smith *et al*., 2011; Pyron *et al*., 2015). Thirdly, the morphological convergence hypothesis postulates that morphological similarity among cryptic species may evolve independently due to similar selection pressures (Stayton, 2006; Bravo, Remsen & Brumfield, 2014). Whatever the underlying cause, morphological similarities within cryptic species complexes and inter‐observer biases make it incredibly difficult for scientists to identify and distinguish among unique taxa (Ellis *et al*., 2006; Jörger and Schrödl, 2013; Schüßler *et al*., 2024) and recognise true biodiversity within communities of organisms (Beheregaray & Caccone, 2007). Many cryptic species have therefore remained, and in many cases still remain, undetected (Walters *et al*., 2021).

### (3) Why do many cryptic species remain undetected?

Due in part to cryptic species complexes, it is hypothesised that most of the world's estimated 5–30 million species are undescribed (Stork, 1993; Liu *et al*., 2022). Many cryptic species have remained undetected for so long due to the way in which taxonomists and field biologists conducting biodiversity inventories classify the taxa that they encounter (Jörger & Schrödl, 2013). As humans, we rely heavily on visual cues to process information, and traditional species classification techniques have relied predominantly on an organism's appearance and morphological characteristics (Bickford *et al*., 2007). This is likely due to the nature of taxonomical collections, where phenotypes based on other biological traits such as behaviour are less readily recognisable in the fixed material. The true biodiversity of morphologically indistinguishable species has therefore mostly been overlooked historically, and groups of taxa have been incorrectly classified as single nominal species (de León & Nadler, 2010; Adams *et al*., 2014).

Biologists now recognise that morphological traits alone are not sufficient to differentiate cryptic species from one another (Jörger & Schrödl, 2013). The recent advancement of genetic and molecular approaches has allowed scientists to classify and identify species whose identities were previously unknown, or incorrectly mistaken for closely related taxa more accurately (Witt, Threloff & Hebert, 2006; Fišer *et al*., 2018). This has been further supplemented by the study and utilisation of reproductive isolation mechanisms, such as species‐specific mating calls and pheromones, which can be applied outside of the laboratory to identify cryptic species of insects (Tishechkin, 2014), amphibians (Wang *et al*., 2017), primates (Braune, Schmidt & Zimmermann, 2008), bats (Ramasindrazana *et al*., 2011), and birds (Freeman & Montgomery, 2017) relatively reliably *in‐situ*. Even more recently, machine learning and artificial intelligence (AI) techniques have become commonplace in many fields of biological study, and such computer‐based approaches are now also being applied to solve cryptic species identification problems with high levels of precision (e.g. Derkarabetian, Starrett & Hedin, 2022; Pinho *et al*., 2023). While some philosophers still argue about what constitutes a species (Donoghue, 1985; Mayr, 1996; Wang *et al*., 2020), integrative taxonomical approaches now use a combination of molecular, phenological, biogeographical, ecological, anatomical, and behavioural data, in addition to subtle morphological discrepancies where possible, to delimit species boundaries (Dayrat, 2005; Padial *et al*., 2010).

### (4) Taxonomic expansion due to cryptic species detection

**Fig. 1:**
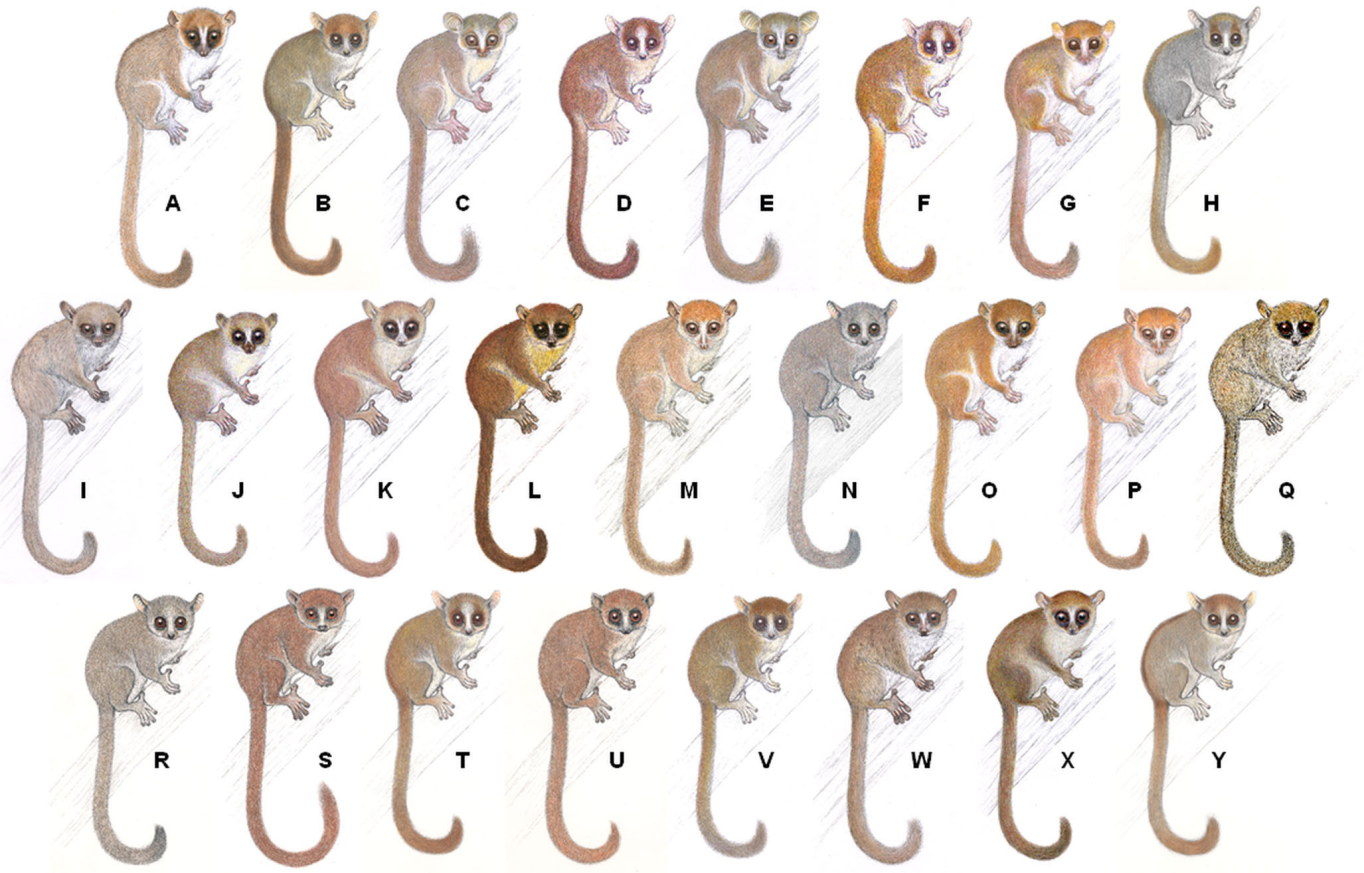
The mouse lemurs (genus *Microcebus*) are a cryptic species complex of 25 species (Schüßler *et al*., 2020, but see van Elst *et al*., in press), once considered to be a single nominal species (*M. murinus*). *Microcebus* spp. are almost morphologically indistinguishable from each other but can be identified by subtle discrepancies in pelage colouration (exaggerated in this figure) and their acoustic signals. A, *M. arnholdi*; B, *M. berthae*; C, *M. bongolavensis*; D, *M. borahi*; E, *M. danfossi*; F, *M. ganzhorni*; G, *M. gerpi*; H, *M. griseorufus*; I, *M. jollyae*; J, *M. jonahi*; K, *M. lehilahytsara*; L, *M. macarthurii*; M, *M. mamiratra*; N, *M. manitatra*; O, *M. margotmarshae*; P, *M. marohita*; Q, *M. mittermeieri*; R, *M. murinus*; S, *M. myoxinus*; T, *M. ravelobensis*; U, *M. rufus*; V, *M. sambiranensis*; W, *M. simmonsi*; X, *M. tanosi*; Y, *M. tavaratra*. Illustrations copyright 2013 Stephen D. Nash/IUCN SSC Primate Specialist Group, used with permission.

As a consequence of the increased use of integrative taxonomical approaches since the 2000s (Dayrat, 2005), countless nominal species have been found to comprise distinct reproductively isolated taxa (Chenuil *et al*., 2019). The detection, description, and scientific classification of these cryptic species has resulted in rapid taxonomic expansion of many groups of organisms, and an explosion of newly described taxa (Bickford *et al*., 2007; Funk, Caminer & Ron, 2012). Among plants, examples of cryptic species complexes exist in bryophytes (Renner, 2020), ferns (Wei *et al*., 2022), mahogany trees (Cavers *et al*., 2013) and arctic angiosperms (Grundt *et al*., 2006), whilst numerous examples are known from fungi (e.g. Crespo & Lumbsch, 2010). In animals, cryptic diversity is particularly profound in invertebrates such as in terrestrial spiders (Tyagi *et al*., 2019), moths (Lassance *et al*., 2019) and ants (Seifert, 2009), and in zooplankton (Sáez & Lozano, 2005), amphipods (Witt *et al*., 2006) and urchins (Egea *et al*., 2016) in marine environments. However, cryptic species complexes also frequently occur in vertebrates, such as in birds (Freeman & Montgomery, 2017; Taylor *et al*., 2019), bats (Ramasindrazana *et al*., 2011; Juste *et al*., 2018), frogs (Funk *et al*., 2012; Chan *et al*., 2020), lizards (Smith *et al*., 2011; Pinho *et al*., 2023), geckos (Pearson *et al*., 2007; Kotsakiozi *et al*., 2018) fish (Hyde, Underkoffler & Sundberg, 2014; Parmentier *et al*., 2022), and prosimian primates (Braune *et al*., 2008; Pozzi *et al*., 2020; Fig. 1).

While the validity of many new species is hotly contested, and in some cases regarded as unnecessary inflation (Tattersall, 2007; Trontelj & Fišer, 2009), the importance of their recognition is paramount. This is so that (*i*) future taxonomic confusion can be avoided, (*ii*) true species richness and biodiversity is accurately quantified, and (*iii*) conservation applications focus on individual species, or species complexes, that most urgently require attention (Bickford *et al*., 2007; de León & Nadler, 2010; Chenuil *et al*., 2019). Further, the acknowledgement of the existence of cryptic species is also important for disease diagnosis and prevention (de León & Nadler, 2010, 2016), biological control (Smith *et al*., 2018), and the treatment of envenomation in human populations (Sanders, Malhotra & Thorpe, 2006). Cryptic species complexes are now known to occur throughout the world, in both terrestrial and aquatic environments, posing serious conservation challenges.

### (5) Why care about cryptic species?

The primary conservation concerns of non‐biologists are often associated with charismatic and visually unique organisms: these traits are not present in cryptic species (Macdonald *et al*., 2015). On the other hand, many biologists prioritise the protection of keystone species and other taxa with profound ecosystem importance (Myers *et al*., 2000). Indeed, members within cryptic species complexes sometimes fill the same niches and ecological roles. This poses the question: why should we care about cryptic species? Many cryptic taxa are of great ecological, economical, and even medical importance (Bickford *et al*., 2007). Overlooking, misidentifying, and most importantly, the extinction of cryptic species can therefore have serious consequences for ecosystem function, management, and disease control. Therefore, it is important to protect and conserve these species so that their ecological and economic value is not lost (Chadès *et al*., 2008). The recognition of as‐yet undescribed species also has equal value; new species may be important for solving medical and food security problems in the future (e.g. de León & Nadler, 2010; Ji *et al*., 2020), and efforts to reveal cryptic diversity are therefore justified. We must also consider that most of todays' distinctive and charismatic species started their evolution as morphologically similar taxa, so ignoring cryptic species could lead to a reduced potential for new non‐cryptic species to evolve. Due to (*i*) the severity of extinction risk among vertebrates (Ripple *et al*., 2017), and (ii) our poorer collective understanding of the conservation status of the world's invertebrates (Cardoso *et al*., 2011), the following sections of this paper will focus primarily on cryptic vertebrates.

## II. CHALLENGES IN CONSERVATION OF CRYPTIC SPECIES

### (1) Ongoing threats to global biodiversity

Cryptic species are threatened by the same anthropogenic processes that imperil all organisms, including (but not limited to) the ongoing destruction, fragmentation and degradation of natural habitats, land‐use change, overhunting, and unmitigated climate change (Maxwell *et al*., 2016; Tilman *et al*., 2017). In the literature, habitat loss/degradation and invasive species introductions have been identified as the two single greatest drivers of species extinctions, and arguably the greatest current and future threats to biodiversity (Brooks *et al*., 2002; Pimm *et al*., 2006; but see Bellard, Marino & Courchamp, 2022). Both are highly relevant for cryptic species. Habitat loss and invasive species introductions are particularly pronounced in biodiversity hotspots, characterised by high levels of floral and faunal biodiversity and endemicity (Myers *et al*., 2000). It has been speculated that cryptic species numbers may be higher in tropical biodiversity hotspots than in other areas, although this has yet to be systematically studied (Bickford *et al*., 2007). As the most species‐diverse habitat on Earth, tropical forests are the most notable biodiversity hotspot example, as they harbour approximately 50% of the world's terrestrial plant and animal taxa and are crucial habitats for almost five million animal species (Lovejoy, 1997; Wright, 2005). However, tropical forests are being lost at an alarming rate, which poses immense problems for the species that inhabit them (McFarland, 2018). Islands are another important habitat when considering cryptic species. Due to their geographic isolation, islands are important hotspots of endemism (Kier *et al*., 2009), yet island biodiversity is often susceptible to invasive species introductions by human populations (Pimm *et al*., 2006). Due to their importance as a habitat, all global biodiversity hotspots have received significant conservation attention (Brooks *et al*., 2006), and protected area systems and species‐specific action plans have been established to safeguard the future of the most threatened populations (Myers *et al*., 2000). However, the successful conservation of cryptic species often poses additional challenges (Hey *et al*., 2003; Bickford *et al*., 2007), which I highlight below.

### (2) Recent taxonomic description

The first conservation challenge for many cryptic taxa relates to their recent taxonomic description. Due to the aforementioned historical reliance on morphological traits to identify species, many cryptic species have only been described since the application of modern molecular and integrative taxonomical approaches to species classification became commonplace in the early 2000s (Dayrat, 2005; Padial *et al*., 2010; Jörger & Schrödl, 2013; Fišer *et al*., 2018; Struck *et al*., 2018). The splitting of nominal species into multiple cryptic species creates immediate problems for conservation biologists. Nominal species may have been spread over large geographic areas, whereas the newly described taxa within the resultant cryptic species complexes often have comparatively restricted ranges and smaller populations (Cebbalos & Ehrlich, 2009; Morrison *et al*., 2009), and they would therefore be at a much greater risk of extinction (Liu *et al*., 2022). Further, conservationists often have little (or no) data on the ecology, behaviour, and habitat requirements of these newly described species (Tapley *et al*., 2018); they would have received limited research and conservation attention. Considering that approximately 60% of new vertebrate taxa described since 1990 are cryptic species (Cebbalos & Ehrlich, 2009; Liu *et al*., 2022), the challenge that this poses for successful species conservation becomes obvious. Taxonomic revisions of a wide variety of vertebrates have been undertaken in recent years, examples of which include giraffes (Fennessy *et al*., 2016), mouse lemurs (Schüßler *et al*., 2020; van Elst *et al*., in press), giant salamanders (Yan *et al*., 2018), and scaly‐toed geckos (McDonald *et al*., 2022). Further, some recently described species such as the Tapanuli orangutan, *Pongo tapanuliensis* (Wich *et al*., 2019) and the Skywalker hoolock gibbon, *Hoolock tianxing* (Fan *et al*., 2017) are among the most threatened species worldwide.

### (3) Data deficiency

A second conservation challenge for cryptic species relates to deficiencies of robust empirical data. To assess accurately the conservation status of a species, an informed understanding of their distribution, population dynamics and habitat requirements is essential (Syfert *et al*., 2014; Tapley *et al*., 2018). This is because such spatial and ecological data are paramount to assign species a formal classification status on the IUCN *Red List* (Hoffmann *et al*., 2008; Rodríguez *et al*., 2011), which is a vital tool for assigning conservation priorities, assessing extinction risk, and measuring short‐ and long‐term impacts on threat status (Collen *et al*., 2016; Betts *et al*., 2020). Due to the recent taxonomic description of many cryptic species, scientists simply do not have adequate data to make an accurate assessment of their current conservation status (Tapley *et al*., 2018). Approximately 14% of all assessed species (~20,000 animal, plant, and fungi taxa) are currently classified as Data Deficient, and even among vertebrates, many newly described species of fish, amphibians, and reptiles have out‐of‐date species assessments or have yet to be formally assessed (Cazalis *et al*., 2022: Fig. 2). Coarse assessments of Data Deficient species may hinder the conservation of these taxa (Hermoso & Kennard, 2012), and so conservation biologists are striving to obtain the necessary data to facilitate their reliable classification (Roberts, Taylor & Joppa, 2016). However, data acquisition is time‐consuming and financially costly (Hermoso, Kennard & Linke, 2013), and is especially difficult for cryptic species that are hard to identify and study in their natural habitats (Claridge *et al*., 2004; Aubry *et al*., 2017).

**Fig. 2:**
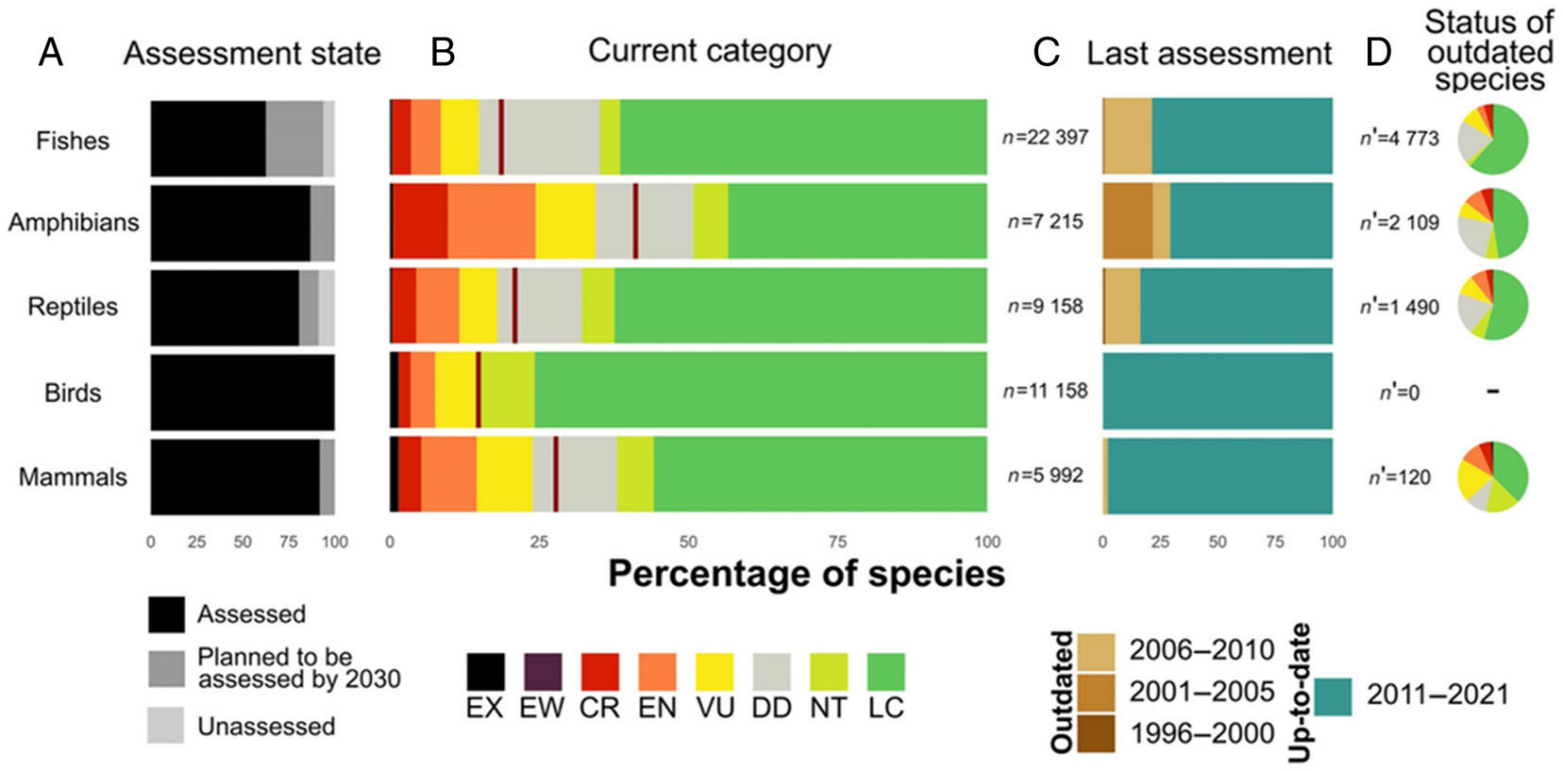
IUCN *Red List* assessment information for the five vertebrate groups as of 2022. Figure obtained and modified from Cazalis *et al*. (2022), used with permission. CR, Critically Endangered; DD, Data Deficient; EN, Endangered; EW, Extinct in the Wild; EX, Extinct; LC, Least Concern; NT, Near Threatened; VU, Vulnerable.

### (4) Sympatric species

A third challenge for the conservation of cryptic species relates to taxa that live in sympatry (species with overlapping geographic ranges). Global biodiversity hotspots can harbour hundreds of different species that live in close proximity to one another within the same habitat type (Myers *et al*., 2000). For most species, their unique morphology, appearance, or features make them easy to detect and identify visually in the field (Macdonald *et al*., 2015), and this enables scientists to locate, monitor and conserve populations of these taxa more efficiently. By contrast, sympatric cryptic species cannot be discriminated from each other as easily, and delays in their detection and field identification can hamper conservation efforts for their populations (Bickford *et al*., 2007; Chadès *et al*., 2008). As many closely related species occupy the same or very similar ecological niches, and are often competing for the same resources, cryptic species should theoretically not be able to co‐exist within the same geographic range (Zhang, Lin & Hanski, 2004; Violle *et al*., 2011). However, significant niche partitioning occurs within many cryptic species complexes (e.g. Ashrafi *et al*., 2011; Scriven *et al*., 2016; Hending *et al*., 2023), and cryptic species can coexist together in a wide variety of habitats (Zhang *et al*., 2004). Sympatry among cryptic species is therefore more frequent than originally anticipated (Scriven *et al*., 2016; Delić *et al*., 2023). Examples of sympatric cryptic species can be found in bat communities (Razgour *et al*., 2011), frogs of the Amazon (Funk *et al*., 2012), Tegu lizards (Murphy *et al*., 2016), and high‐altitude specialist dwarf lemurs (Blanco *et al*., 2008), and many of these sympatric cryptic species groups encompass taxa now listed as threatened on the IUCN *Red List*.

### (5) Socioeconomic challenges and conservation priorities

Hotspots for biodiversity and cryptic species tend to be located in the Global South where there is less funding available for scientific research (Myers *et al*., 2000). Species surveys and applied conservation, especially using integrative taxonomical approaches that may be expensive, requires money that institutions and governments may not have, nor want to dedicate to scientific endeavours when human welfare and food security issues are a priority (Chan *et al*., 2007). The conflict between economic issues and conservation presents another challenge for the future study of cryptic species. Perceptions of cryptic species complexes may provide further hinderance. While species within these complexes may perform different roles within the ecosystem and fill distinct ecological niches (Ashrafi *et al*., 2011; Scriven *et al*., 2016), the non‐conservationist audience (including the general public and government employees) may not be aware of the importance, or even existence, of cryptic species. More attention is given to charismatic species and lesser‐known cryptic taxa are often overlooked (Macdonald *et al*., 2015). As a substantial portion of funding for nature conservation projects comes from either public donations or government funds (Anyango‐van Zwieten, Lamers & van der Duim, 2019), there is increased pressure to target the species that people “care about the most” (Colléony *et al*., 2017). When conservation scientists have funding for projects targeting cryptic species complexes, there is also the challenge of deciding which members of that complex to target. Data deficiency can prevent conservationists from making informed decisions (Tapley *et al*., 2018), which may result in misallocation of financial resources and jeopardising of conservation (Waldron *et al*., 2013).

## III. OVERCOMING THE CHALLENGES

### (1) Cryptic species recognition

The recognition of cryptic species is an important first step in overcoming the difficult challenge of cryptic species conservation. Firstly, recognition is meant in the literal sense; although not something to be trivialised (Trontelj & Fišer, 2009), conservation researchers and practitioners need to acknowledge and recognise the phenomenon of cryptic diversity (Hey *et al*., 2003; Cook, Page & Hughes, 2008). The formal process of naming and describing a cryptic species is a link often missing between taxonomical science and applied research, yet this is an important aspect that turns molecular trees into products for other biologists and conservationists (Delić *et al*., 2017). They must also consider cryptic species when planning studies, biodiversity surveys, and conservation interventions in order to capture the full taxa spectrum (Bickford *et al*., 2007; Bessey *et al*., 2023). Many past surveys of biodiversity and species community composition either (*i*) have used methods unsuitable to detect cryptic species (over‐reliance on visual identification methods) or (*ii*) have not considered the possibility that cryptic species may be present (Feckler *et al*., 2014). For studies and surveys conducted prior to the 1980s, this can be attributed to two factors. Firstly, there was simply limited knowledge of cryptic species, and whilst researchers were aware of their existence, there had been little scientific effort to disentangle cryptic species complexes (Bickford *et al*., 2007; Fišer *et al*., 2018; Chenuil *et al*., 2019). Secondly, the primary means to detect, recognise and describe cryptic taxa is genetic analysis, particularly the polymerase chain reaction (PCR) (Saiki *et al*., 1988), which was only introduced in the mid‐1980s. As cryptic diversity was only just being revealed, complete and accurate species assessments could not have been conducted at this time, to no fault of the conservationists endeavouring to protect wild populations. However, many species assessments that have occurred in global biodiversity hotspots over the last 30 years have continued to rely primarily on visual taxonomic identification (Beheregaray & Caccone, 2007; Pearman *et al*., 2016), resulting in underestimates of true biodiversity, and consequently misleading or misinforming resultant conservation interventions (Vieites *et al*., 2009; Adams *et al*., 2014; Huntley *et al*., 2019). To overcome this first challenge, it is therefore imperative for conservation scientists to recognise the existence of cryptic diversity, acknowledge missed opportunities and mistakes (due to both a lack of knowledge or poor planning) in previous studies, and apply this information to future work (Feckler *et al*., 2014; Black, 2020).

### (2) Cryptic diversity/biodiversity

Secondly, in the context of taxonomic identification, how do we overcome of the challenge of identifying cryptic species *in situ*? Here, I summarise how we can detect this cryptic diversity in the field, using an integrative approach.

#### (a) Morphometrics

Morphometric analysis and comparison among closely related taxa is a particularly useful way in which cryptic species can be discriminated from one another in the field. Unique morphological traits, many of which are often subtle (e.g. Crottini *et al*., 2011; Arntzen *et al*., 2013), have historically been the most reliable way to identify species of invertebrate that would otherwise be visually indistinguishable (Huber, 1998). The same is true for many vertebrate species, and close inspection and scrutiny of a wide range of body parts can allow scientists to discriminate species from other organisms that at first glance appear identical (Ashrafi *et al*., 2010). Morphometric discrimination has been applied to several cryptic species groups, and such examples include differentiation among bats *via* wing shape and nose leaf (Furman *et al*., 2010; Chornelia *et al*., 2022), identification of cryptic amphibians by their external gland position and appendage morphology (Arntzen *et al*., 2013; Majtyka *et al*., 2022), and scalation in geckos (Crottini *et al*., 2011). Due to high intra‐species variation, it is worth noting here that colour patterns are rarely reliable means of discriminating among any species, cryptic or non‐cryptic (e.g. Carolan *et al*., 2012). Despite its accuracy, the usefulness of morphometric analysis in rapid biodiversity assessments is limited, as its application is restricted primarily to species that are either slow‐moving, ground‐dwelling, small‐bodied or otherwise easy to handle and capture (Hoffmann *et al*., 2010). Unless specialised equipment such as dart‐guns, live‐traps, or mist‐nets are employed (which are costly and highly intrusive), cryptic species that are large‐bodied, arboreal or volant cannot easily be captured, handled, and identified in the field. Further, some unique morphological features are so subtle that they can only be detected under a microscope (Simo‐Riudalbas *et al*., 2017); this is often not a practical field method in rapid biodiversity assessments. Such species cannot be efficiently identified by their morphology alone, and so other means of recognising these species are required.

Whilst *in‐situ* morphometric identification is challenging and not always possible, it must be stated that morphological approaches are still required to confirm that two superficially similar specimens really are cryptic for formal species descriptions. Form is the most important constituent of diversity and disparity, even at the molecular level, and morphological discrepancies are (usually) the most reliable pointer to biological difference.

#### (b) Reproductive isolation mechanisms

Many cryptic animals use loud calls for spacing and sexual advertisement. These calls serve as important reproductive isolation mechanisms, enabling these species to differentiate between hetero‐ and conspecifics (Braune *et al*., 2008; Ramasindrazana *et al*., 2011; Wang *et al*., 2017). Such loud calls can be passively detected and recorded by field researchers from long distances without the need for capture, and bioacoustic surveys are highly useful for rapidly detecting and identifying vocal cryptic species (Lambert & McDonald, 2014; Smith *et al*., 2020). Due to technological advances, bioacoustics recording devices have become evermore affordable in the last 10–20 years, and field biologists can now choose from a variety of high‐quality, efficient, and customisable options when planning biodiversity assessment expeditions (e.g. Hill *et al*., 2018; Wijers *et al*., 2021). Freely available call catalogues (Kasten *et al*., 2012), a variety of acoustic analysis software (Mcloughlin, Stewart & McElligot, 2019), and machine learning classifiers (Bianco *et al*., 2019) are also now widely used, and these enable biologists efficiently to isolate signals of interest from background noise and identify calls to species level with minimal analysis effort (Lambert & McDonald, 2014). In addition to taxa or group‐specific survey approaches [e.g. bats (Ramasindrazana *et al*., 2011), frogs (Measey *et al*., 2017), birds (Freeman & Montgomery, 2017)], bioacoustics surveys can also be used to quantify overall biodiversity at the soundscape level (Rajan *et al*., 2019; Alcocer *et al*., 2022), and acoustically detectable species, whether they are cryptic or not, are reliable indicators of total species richness (Smith *et al*., 2020). Acoustic approaches are therefore highly useful to record and quantify overall biodiversity, even if all species present in an area are not detected. Unfortunately, there are many examples of cryptic species that are both difficult to capture and non‐vocal, and detection and identification of these elusive taxa via morphological and/or passive acoustic means is near‐impossible [e.g. primates (Zimmermann, 1995); rodents (Thomas *et al*., 2020); arboreal reptiles (Nordberg & Schwarzkopf, 2015)]. So how can we efficiently detect these species in biodiversity assessments so that true species richness is reflected in the results?

#### (c) Camera traps, environmental DNA, and other methods

Both active and passive survey methods often miss certain species due to their behaviours or due to difficulties in accessing their habitats and ecological niches (Dorcas & Willson, 2009; Nordberg & Schwarzkopf, 2015), and this is especially true of elusive, non‐vocal animals. Whilst many taxa that fit this description were likely missed in biodiversity surveys that occurred in the last century, scientists now go to great lengths to detect these species so that true overall diversity values are captured, and a number of new, innovative techniques have been developed in recent years to overcome this challenge (Fišer *et al*., 2018; Table 1). Camera traps are useful to detect the occurrence of cryptic species, particularly mammals, in remote and difficult‐to‐reach areas (Harley *et al*., 2014; Thomas *et al*., 2020). Accurate identification keys and automated classifiers now allow for the identification of morphologically cryptic species from their images alone without the need for capture (Falzon *et al*., 2019; McKibben & Frey, 2021; Pinho *et al*., 2023). Although expensive, collection and analysis of environmental DNA (eDNA) can reveal the presence of cryptic species that are difficult to see or hear and therefore near‐impossible to detect *via* other methods (Thomsen & Willerslev, 2015). eDNA is a particularly useful indicator in aquatic systems, but some terrestrial biodiversity studies have made use of it *via* artificial structures attractive to target species groups (e.g. snakes, lizards), and such methods often yield higher diversity estimates than conventional survey techniques (Beng & Corlett, 2020; Matthias *et al*., 2021; Ruppert *et al*., 2022). Cryptic species detection has also been achieved through some more‐unusual, innovative, and unorthodox methods, including detection dogs (Thomas *et al*., 2020) and thermography (Karp, 2020), whilst indigenous knowledge can also be utilised. High‐throughput sequencing is an additional alternative that can be more efficient and cost‐effective than traditional, labour‐intensive biodiversity survey techniques (Ji *et al*., 2013). In summary, an integrated, holistic approach is needed to (*i*) determine the presence of all species at a site and (*ii*) to quantify an overall biodiversity value at a specific location.

**Table 1:** A summary of survey techniques that enable the detection and/or identification of cryptic species *in‐situ*. The listed techniques and their use cases, advantages and disadvantages is not exhaustive, and does not include techniques that enable cryptic species detection in non‐field‐based environments [e.g. captured specimens in a laboratory, using software/artificial intelligence (AI), etc.].

Detection method	Primary use cases	Primary advantages	Primary disadvantages
Acoustic surveys	Taxa that use acoustic signalling for spacing/advertisement, or as a reproductive isolation mechanism.	Non‐invasive. Not effort‐intensive (passive/remote). Can cover large area.	Limited to vocally active taxa. Equipment often expensive.
Camera traps	Elusive and rare taxa sensitive to anthropogenic activity.	Non‐invasive. Not effort‐intensive (passive/remote). Can cover large area.	Detection limited by resolution detail (detected species needs to be close to camera). Equipment often expensive. Often unsuitable for small species.
Detection dogs	Elusive and hard‐to‐detect taxa.	Enables *in‐situ* identification. No equipment required.	Covers small area. Expensive to train dogs.
Environmental DNA	Aquatic taxa, and terrestrial taxa with predictable travel routes (direct contact with capture medium required).	Non‐invasive. Not effort‐intensive (passive/remote). Applicable to any taxa.	Over‐reliance on animal–capture medium contact. Very expensive.
Morphometric analysis	Taxa with subtle morphological differences, or structural reproductive isolation mechanisms (genital morphology).	Enables *in‐situ* identification. Little equipment required.	Limited to easy‐to‐capture, non‐arboreal/volant, non‐elusive species. Time‐consuming. Invasive and stressful for animal.
Thermography/drones	Hard‐to‐detect species of sufficient size.	Non‐invasive. Can cover large area. Works in all habitat types.	Only facilitates localisation – other methods needed for species identification. Can be expensive.

### (3) Filling knowledge gaps

The recognition and detection of cryptic species is a notable challenge (de León & Nadler, 2010), and so conservation of their populations is an even more difficult endeavour (Hey *et al*., 2003; Bickford *et al*., 2007). The enormity of this challenge often raises the question of whether further survey and conservation investment into cryptic species is actually worthwhile (Chadès *et al*., 2008). Fortunately, the methods summarised above have enabled scientists to start to disentangle some cryptic species complexes and begin to overcome the challenges of recognition and detection (Melville *et al*., 2014). This information, if utilised correctly, can provide us with important information to facilitate the future study and conservation of cryptic species (Boitani *et al*., 2011). Detection of cryptic species *via* integrative and holistic approaches enables field biologists to build up databases of their occurrence points, which can then be used to create ecological niche models (ENMs) and species distribution models (SDMs) (Peterson, 2001; Murphy & Smith, 2021). In recent years, ENMs and SDMs have become crucial tools for understanding the geographic ranges and habitat requirements of cryptic species (e.g. Pearson *et al*., 2007; Schüßler *et al*., 2023), the environmental and anthropogenic factors that influence their occurrence (e.g. Hending *et al*., 2023), and changes in the conservation and threat status of species listed as threatened on the IUCN *Red List* (e.g. Thorn *et al*., 2009; Hending, 2021). As freely available environmental data and low numbers of occurrence points are the only requirements to generate accurate and reliable ENMs and SDMs for exploratory analysis (Wisz *et al*., 2008), both model types are paramount to expanding our knowledge on understudied and recently described cryptic species (Sattler *et al*., 2007; Aubry *et al*., 2017; Martin *et al*., 2022), and assigning IUCN statuses to species currently listed as Data Deficient or Not Evaluated.

ENMs and SDMs can be utilised to fill knowledge gaps in three primary ways. Firstly, both methods can predict the fundamental niche area of a species, from which their geographic range, distribution, and extent of occurrence can be discerned (Pearson *et al*., 2007). These data are vital to developing global conservation strategies (Boitani *et al*., 2011), and form the basis on which species are assessed and classified on the IUCN *Red List* (Hoffmann *et al*., 2008; Cazalis *et al*., 2022). These data can be supplemented with additional information on the species' ecology, behaviour and habitat usage to form predictions of realised niche area and areas of occupancy (Marcer *et al*., 2013; Breiner *et al*., 2017), however such data are limited for many understudied cryptic species. The second application of ENMs and SDMs is to guide the future search for cryptic species (Fois *et al*., 2018). There are often minimal occurrence points for elusive and understudied cryptic species, and it is therefore of vital importance to identify areas where these taxa may persist so that they can be surveyed for at these locations in the future (Ferrer‐Sánchez & Rodríguez‐Estrella, 2016; Fois *et al*., 2018). Such information also pinpoints areas of high biodiversity if this approach is adopted for multiple organisms, and consequently this facilitates conservation planning (Fleishman, Noss & Noon, 2006; Heberling *et al*., 2021). The identification of these occurrence areas may also enable scientists to revisit samples and taxonomic collections from specific field sites; specimens of cryptic taxa could be revisited to check if they were identified and labelled correctly based on their morphometrics and field characteristics. Thirdly, careful analysis of ENMs and SDMs can reveal the most prominent climatic, habitat‐related, and anthropogenic drivers of cryptic species distribution. Whilst this lends support to achieving the desired points relating to the first two applications, this information additionally facilitates differentiation among the geographic range and habitat preference of sympatric species, and provides first insights into the ecology of as‐yet unstudied and recently described cryptic taxa (Cavalcante *et al*., 2020; Hending *et al*., 2023).

## IV. FUTURE DIRECTIONS

As outlined above, innovative survey methods and recent advancements in hardware and software enable scientists to detect cryptic species and study their demography and ecology more easily now than ever before. Due to difficulties in implementing effective conservation strategies for cryptic taxa in comparison to more‐recognisable, detectable, and charismatic/keystone species, the way in which we apply this new knowledge is crucial for effective conservation action to take place. This section outlines some points that will likely be of vital importance for achieving effective conservation of cryptic species in the future.

### (1) Remote sensing

Remote sensing methods are increasingly common in biodiversity surveys as hardware and software becomes more affordable and reliable (Turner *et al*., 2003; Hill *et al*., 2018; Wijers *et al*., 2021). Although remote sensing approaches are applicable to all taxa, and whilst some are only suitable for certain species (e.g. seismic sensing for large‐bodied, easily recognisable animals; Szenicer *et al*., 2022), such methods are often the only effective means of revealing the presence of cryptic organisms. Acoustic surveying is a particularly useful method for cryptic species detection (Lambert & McDonald, 2014; Smith *et al*., 2020), and its advantages enable surveying of large areas for many different groups of vocally active taxa, particularly those that are most‐reliably identified by their loud calls (reproductive isolation mechanisms; Braune *et al*., 2008). In addition to its usefulness in determining presence and distribution, bioacoustic surveying now also enables the assessment of species habitat usage and preference (Hending *et al*., 2020) and population density calculation (Marques *et al*., 2013), which is vital data for *Red List* conservation assessments (Boitani *et al*., 2011; Santini *et al*., 2019). Analysis at the soundscape level can provide useful proxies of overall species richness, and therefore suggest the existence of undetected cryptic taxa that may have been missed by other survey methods; acoustically detectable species often reflect total biodiversity (Smith *et al*., 2020). Many global regions with high biodiversity levels are heavily impacted by anthropogenic disturbance (Myers *et al*., 2000), and scientists are therefore striving to make biological studies as non‐invasive as possible (e.g. Gruber & Wood, 2022). Remote sensing methods such as acoustic surveying and camera trapping are therefore preferable for cryptic species detection and identification in comparison to more hands‐on techniques (trapping, capturing, morphometric analysis, etc.) that cause stress to animals and disturbance and degradation to natural habitats. Most remote sensing techniques are also passive, and they enable researchers to carry out additional methods in parallel (transects etc.) to maximise survey effort and increase chances of cryptic species detection. Due to these obvious advantages, remote sensing will surely play a vital role in cryptic species surveys, identification, and detection in the future, and such methods will be vitally important for achieving successful conservation of cryptic species (Turner *et al*., 2003; Nagendra *et al*., 2013).

### (2) Holistic biodiversity monitoring

To achieve cryptic species detection and conservation, biodiversity scientists urgently require a deeper understanding of complex, interconnected ecosystems and their faunal and floral richness (Bickford *et al*., 2007). This requires a range of approaches, such as those outlined above, to acquire a variety of data types to reduce uncertainties in the inference of field data and decision‐making in species identification (Gibb *et al*., 2019; Wijers *et al*., 2021). Observations of informative signals and subsequent analyses and models are biased by a mix of human senses and dictated by equipment/instrument availability, and this may result in us misinterpreting or missing valuable cues. In the case of cryptic species, the informative signals or cues are made up of the subtle traits that enable us to discriminate among closely related taxa (Dayrat, 2005; Padial *et al*., 2010; Arntzen *et al*., 2013). As the most important indicators for the existence of cryptic, previously undetected taxa, these cues need to be identified as early and as efficiently as possible for accurate diversity assessments and subsequent implementation of conservation actions (Bickford *et al*., 2007).

Holistic, large‐scale, remote and non‐invasive surveys and monitoring programs will likely be paramount to elucidating the broad‐scale demographic and ecological information required for successful conservation of all species, including cryptic taxa, in the future (Picciulin *et al*., 2019; Hausdorf, 2021). However, whilst methodological innovation and the availability of low‐cost equipment continues to progress, some technological challenges prevent scaling up of localised surveys to broad‐scale, long‐term monitoring endeavours. Integrative and multi‐method species surveys that are both spatially and temporally large, particularly those that utilise remote sensing, produce massive data sets; these are problematic to store, process, and extract useful information (cryptic species cues) from. Automated processing pipelines therefore need to be put in place to deal with such data sets (Szenicer *et al*., 2022), and this poses a real barrier for biologists and field researchers who may not be familiar with computer‐ and script‐based data‐crunching techniques (Kumar & Dudley, 2007; Shade & Teal, 2015). Conservation action needs to be efficient, timely, and affordable (Laurance *et al*., 2012), and a transition to rapid online data processing and real‐time species identification, likely reliant on reference libraries (Gibb *et al*., 2019), would be ideal. Although such a shift would rely on highly sophisticated and technologically advanced survey hardware, this would reduce volumes of stored data, improve identification accuracy, and increase the rate at which results are disseminated and translated into applied conservation actions. A combination of holistic, large‐scale, and complementary data sets will be vitally important for disentangling cryptic species complexes (Bickford *et al*., 2007; Jörger & Schrödl, 2013), rapidly identifying taxa to species‐level *in‐situ* and implementing conservation to protect the ever‐growing list of threatened species.

### (3) Collaboration, data sharing, and inclusive science

Little distributional data exist for many cryptic species and there is high ambiguity regarding their conservation status (Tapley *et al*., 2018; Cazalis *et al*., 2022). Due to low sample sizes in cryptic species demographic data sets, it is impossible for conservation actions to be well informed by these uncertainties and lack of data (Roberts *et al*., 2016). As highlighted above, building upon this existing data requires large investment of finances, time, and qualified personnel (Hermoso *et al*., 2013; Buxton *et al*., 2020), yet the sharing of information, resources, and data may enable conservationists to overcome this challenge (Martin *et al*., 2022). Due to a range of geographical, political, logistical, and legislative factors, many species surveys and distributional studies occur over small spatial and temporal areas (Miller *et al*., 2004; Hermoso *et al*., 2013), resulting in a fragmented and disjointed understanding. It is now recognised that large, often‐international collaborations within which data are shared can alleviate this issue (Bodin, 2017). Numerous studies now pool participants and resources from multiple academic institutions, non‐governmental organisations, government bodies, and indigenous communities to share expert knowledge and skills to overcome these issues, with considerable success (Lauber *et al*., 2011; Martin *et al*., 2022; Mulder *et al*., 2023). Scientists can now make their data sets publicly available online *via* repositories, and this has increased accessibility, methodological transparency, and reproducibility (Tulloch *et al*., 2018). Such data repositories allow for the storage of distributional records and survey results that would not be included in published scientific articles or their supplementary files, and pre‐print servers facilitate rapid, pre‐publication data sharing. Many conservation‐ and biodiversity‐themed journals now mandate fully open access publishing, and fee‐waivers are available to scientists from low‐income countries with little research funding. This relatively new approach ensures that results and data sets can be accessed by those in biodiversity hotspot countries, where the highest levels of cryptic diversity are, and where the necessity for conservation action is most critical (Lovejoy, 1997; Myers *et al*., 2000; Wright, 2005; Bickford *et al*., 2007). Field biologists and conservationists must strive to utilise these valuable resources for guidance on their conservation priorities, and to ensure that associated actions are executed efficiently.

### (4) Protected area networks

Protected areas cover almost 16% of the world's land surface, a value predicted to increase to 30% by 2030 (Meng *et al*., 2023). They are therefore one of the most effective and crucial methods for large‐scale global biodiversity conservation (Naughton‐Treves, Holland & Brandon, 2005; Fuller *et al*., 2010; Pulido‐Chadid, Virtanen & Geldmann, 2023). If established and monitored correctly (Rodrigues *et al*., 2004; Fuller *et al*., 2010), protected areas can be highly effective in protecting populations of all organisms that live within them, thus safeguarding the future of highly threatened species (Le Saout *et al*., 2013). Although they are sensitive to stochastic changes (e.g. wild fires, extreme weather events) and reliant upon enforcement mechanisms to maintain their resilience (Wolf *et al*., 2021; Ghoddousi, Loos & Kuemmerle, 2022), it is widely regarded that protected areas are the most important means through which we can protect biodiversity (Fuller *et al*., 2010; Pulido‐Chadid *et al*., 2023), as they can cover large geographic areas, prevent the destruction and degradation of natural habitat tracts that fall within their borders, and depending on the legislation, they protect all species that live within their boundaries. In biodiversity hotspot areas, many thousands of species may reside in protected area networks (Myers *et al*., 2000; Brooks *et al*., 2006). The importance of protected areas will be especially important for the future conservation of cryptic species that would otherwise be more difficult to protect *via* other means. This is firstly because protected area networks are most frequently established to shelter areas of biodiversity value (Le Saout *et al*., 2013; Meng *et al*., 2023); whilst cryptic diversity is not easily quantifiable, measurable biodiversity of identifiable species and biodiversity index values are often accurate representatives of overall species richness (e.g. Smith *et al*., 2020; Alcocer *et al*., 2022). Protected areas therefore safeguard as‐yet undescribed cryptic taxa by default. Also, protected areas provide a valuable opportunity for biologists to conduct research on newly described, unstudied species. Data from such studies can be used to gain insights into the wider distribution of the species, develop targeted survey protocols for future demographical studies (e.g. *via* remote sensing), and facilitate the development of species‐specific conservation action plans for the most threatened taxa (Scott *et al*., 2010).

### (5) Scaling up conservation success

Conservation of cryptic species requires careful planning as further undetected cryptic complexes may exist in taxa that are already highly threatened (Bickford *et al*., 2007). Further, cryptic species become more difficult to detect, recognise, and protect as their populations decline (Chadès *et al*., 2008). As previously highlighted, conservation interventions are informed by ecological and demographic data (Boitani *et al*., 2011), but this is difficult for cryptic taxa for which this information is lacking. The importance of conservation scaling initiatives and flexible, transferable action plans is now widely recognised (Brooks *et al*., 2006; Battista *et al*., 2017), and arguably of particular relevance for cryptic species that are notoriously difficult to protect. Identifying broad, multi‐taxa conservation strategies with known success (e.g. Nicholson & Possingham, 2006), and applying them to cryptic species, may be a viable way to protect their wild populations. Some of the better‐studied threatened cryptic species have already been the subject of conservation success stories [e.g. *Lepilemur septentrionalis* (Bailey *et al*., 2020); *Agalychnis lemur* (Emmett *et al*., 2020)]. Having been described from the same nominal taxa, these species have multiple closely related species, with similar or near‐identical habitat requirements and ecological niches (Zhang *et al*., 2004). Such practices may therefore be replicated in these taxa, and conservation of these species complexes may be achieved by scaling up and transferring these successful methods across their members (e.g. *Amazona* spp.; Wenner, Russello & Wright, 2012). Species‐specific conservation actions plans are often unviable for cryptic species due to a lack of data, and protected area networks are therefore the most suitable method to protect cryptic species on a large scale (Le Saout *et al*., 2013). However, many cryptic species co‐occur in biodiversity hotspot zones with well‐studied, well‐known, charismatic and keystone species (Myers *et al*., 2000), for which conservation action plans can be developed more easily and efficiently. To maximise output and make conservation more equitable, conservation methods of these species should aim to achieve protection and management of the wider habitat and ecosystem, to ensure that “overlooked” cryptic species also benefit from this conservation attention (i.e. umbrella conservation; Runge *et al*., 2019). This is especially relevant for cryptic species that are not the subject of targeted, independent conservation efforts.

## V. CONCLUSIONS

(1) The discovery of cryptic species has rapidly accelerated since integrative taxonomy became commonplace in the early 21st century. Conservationists now face the challenge of ensuring the survival of these newly described, unstudied species, many of which are already highly threatened with extinction.
(2) Whilst cryptic diversity is now widely recognised, the detection and identification of individual taxa *in situ* is challenging, limited by funding, and has historically been challenged by ineffective methodological approaches. This severely hinders the study of their wild populations. Scientists need to overcome this hurdle as detailed demographic and ecological data are required for informed conservation to be successfully achieved.
(3) Fortunately, a range of species identification, survey, and remote sensing techniques have been developed that enable the detection and identification of cryptic species within their natural habitat, and these protocols have greatly facilitated the study of some species.
(4) Nonetheless, many cryptic species remain unstudied despite the pressing need for conservation intervention, and this lack of data means that species‐specific conservation efforts are therefore not possible for many cryptic species complexes.
(5) Future conservation and monitoring of cryptic species populations will therefore likely be dependent on (*i*) large‐scale, holistic biodiversity survey regimes, (*ii*) inclusive and collaborative conservation efforts through which data are openly available and shared, and (*iii*) habitat and landscape‐scale management and conservation initiatives, such as protected area networks.
